# Trajectories of Cognitive Impairment in Adults Bearing Vascular Risk Factors, with or without Diagnosis of Mild Cognitive Impairment: Findings from a Longitudinal Study Assessing Executive Functions, Memory, and Social Cognition

**DOI:** 10.3390/diagnostics12123017

**Published:** 2022-12-02

**Authors:** Glykeria Tsentidou, Despina Moraitou, Magdalini Tsolaki, Elvira Masoura, Vasileios Papaliagkas

**Affiliations:** 1 Laboratoty of Psychology, Department of Experimental and Cognitive Psychology, School of Psychology, Aristotle University of Thessaloniki, 541 24 Thessaloniki, Greece; 2 Laboratory of Neurodegenerative Diseases, Center for Interdisciplinary Research and Innovation (CIRI), AUTh, 541 24 Thessaloniki, Greece; 3 Greek Association of Alzheimer’s Disease and Related Disorders, 546 43 Thessaloniki, Greece; 4 Department of Biomedical Sciences, School of Health Sciences, International Hellenic University, 574 00 Sindos, Greece

**Keywords:** vascular aging, MCI, executive functions, working memory, episodic memory, ToM abilities, longitudinal study

## Abstract

With the aging of the population, a key concern of both societies and health services is to keep the population cognitively healthy until the maximum age limit. It is a well-known fact that vascular aging has a negative effect on the cognitive skills of adults, putting them at greater risk of developing dementia. The present longitudinal study aimed to evaluate the main dimensions of cognition in two pathological groups with different health profiles: a group of adults with vascular risk factors (VRF) (n = 35) and a group of adults with vascular risk factors and mild cognitive impairment (VRF + MCI) (n = 35). The two groups were matched in age, education, and gender. They were assessed with extensive neuropsychological testing at three different times with a distance of about 8 months between them; the assessment regarded executive functions, memory capacity, and Theory of Mind abilities. The analyses carried out were (a) mixed-measures ANOVA, (b) repeated measures ANOVA, and (c) ANOVA. The findings showed that global cognitive status and short-term memory are the main cognitive abilities that decline in community dwelling people bearing VRF. Hence, this group of adults should be examined at least every 2 years for this decline. As regards people with both VRF and MCI, it seems that the assessment of Theory of Mind abilities can better capture their further impairment. Global cognitive status, task/rule switching function, and long-term memory (delayed verbal recall) were revealed as the abilities that clearly and steadily differentiate VRF people with and without MCI.

## 1. Introduction

The latest scientific data brings to light the close associations among vascular pathology, neuroinflammation, and neurodegeneration, supporting their cooperative effect on the development of neurodegenerative disease with obvious cognitive impairment [1,2]. Vascular aging is defined as the preclinical stage in which changes in the arterial wall are observed including arteriosclerosis, atherosclerosis, and excess vasoconstriction [3]. What we know so far is that vascular pathology can secondarily cause a decline in the patient’s cognition, creating a significantly high risk of later suffering from some form of dementia [4]. Hence, vascular aging differs from healthy aging and indicates the possibility of progression to vascular dementia or Alzheimer’s disease [5,6]; a vascular brain injury is the result of vascular aging in the brain and a potential mechanism that explains the development of cognitive disorders [7]. Several factors contribute to vascular aging; hypertension is an established risk factor for dementia, as it has been observed that both low and high blood pressure values have been associated with cognitive decline in older adults [8,9]. Christian Boctia et al. (2017) have proposed that even orthostatic hypotension is associated with lower processing speed, deficits in visual working memory, and executive functions in persons with Mild Cognitive Impairment (MCI) [10]. Other studies have also supported the relation between chronic low blood pressure and poor results in attention and executive functions [11]. Body mass index, dyslipidemia, a history of smoking and the diagnosis of diabetes are also among the common vascular risk factors [12]. Nishi et al. (2010) have shown a correlation between reasoning ability and reduced glucose reuptake in the right middle frontal gyrus and higher activation in the same area [13]. In 2020, Jung-Min Pyun et al. [12] proposed fibrinogen—a plasma protein—as an indicator of vascular pathology and an active contributor to neurodegenerative diseases, supporting its association with poor performance in attention, executive function, and confrontation naming ability. Tampubolon G. (2016) has also referred to the association between higher levels of fibrinogen and memory impairments [14].

As a step further in a health continuum [15,16], Mild Cognitive Impairment (MCI) is defined as a clinically heterogeneous syndrome with obvious and measurable impairment on one or more cognitive domains [17] with a high risk of progressing to dementia [18]. Given the fact that no successful treatment for dementia has yet been found, any interventions make sense only at this stage or earlier [18] during which cognitive restructuring and neuroplasticity, such as compensation, still apply [19]. Generally, patients diagnosed with MCI display deficits in remembering events, orientation, planning, decision making, and instruction interpreting [20,21]. At the level of neuropathology, it has been shown that adults diagnosed with MCI exhibit amyloid plaques and neurofibrillary tau tangles, specifically in the olfactory cortex, subiculum, and parahippocampal gyrus in the medial temporal lobes [22,23], while at the behavioral level, they have been shown to be mainly disturbed in episodic memory, working memory, and executive functions [24,25,26,27,28].

Considering the heterogeneity of this clinical entity, subdivisions have been created based on the predominant deficits that appear first. Thus, four sub-types have been ‘officially’ formed: single-domain amnestic MCI, multi-domain amnestic MCI, single-domain non-amnestic MCI, and multi-domain non-amnestic MCI [20,29,30]. Recent findings demonstrate that early impairments in episodic memory, executive functions, and working memory constitute markers of progression from mild to severe MCI and from MCI to AD dementia [31,32,33].

More specifically, episodic memory is widely claimed to be a hallmark for the diagnosis of MCI and, also, for the progression to AD dementia [34,35,36]. It has even been argued that the daily challenges of adults diagnosed with MCI are more related to episodic memory capacities [34]. At the neuronal level, disturbances in episodic memory in MCI adults have been associated with cortical thickness, hippocampal atrophy, neuron connectivity, and amyloid-beta measures [37,38,39]. In a very recent study, it has been discussed that delayed recall as a way of assessing episodic memory impairment is one of the most significant predictive factors in defining the progression of Alzheimer’s disease [36]. Regarding the population of adults with vascular aging, research thus far shows findings that tend to be consistent with a decline of episodic memory as well [40,41].

Working memory promotes active short-term maintenance of information for access and manipulation [42]. The association of impaired working memory in MCI sufferers with deficits in selective attention has been extensively discussed [43]. Older adults have trouble retaining more than 4 digits; this decrease in capacity and processing speed is associated with age-related changes in the dorsolateral prefrontal cortex [44]. Several studies [28,45] have argued that working memory is an important predictor of progression from MCI to AD dementia. Regarding impaired working memory in vascular aging, its correlation with vascular risk factors has been explored [46]; adults bearing vascular risk factors had significantly lower performance on binding functions of working memory. The basis of the correlation is given through pathologic changes to the macro- and microvasculature, which could disrupt blood vessel integrity, secondarily causing neuronal injury, structural and functional brain damage. Structural alterations and disruptions of brain connectivity underlie the decline of cognitive skills [47].

As regards deficits in executive functions in MCI, white matter hyperintensities have been proposed as a potential cause [48]. Attention and executive impairments are frequent findings in the neuropsychological examinations of patients with MCI [49]. Often even deficits in executive function, visuospatial skills, and attentional control can be seen before memory deficits [50]. In a 2022 study, it became clear, and it is claimed that task/rule switching abilities are main skills that are touched in MCI and must be evaluated to assess the risk of future dementia [29]. Regarding executive function deficits in adults with vascular aging, an alteration arising in frontal brain regions, a basic structure serving executive functions, has been discussed as an intermediate bridge [51]. In a relevant study, deficits in cognitive planning and cognitive flexibility are recorded in patients diagnosed with incipient vascular dementia differentiating their performance from other related pathological groups [52]. Similar findings were revealed by a cross-sectional study, which showed that adults with vascular pathology present lower performance in specific executive skills (switching/inhibition) [15].

As concerns the Theory of Mind (ToM) and emotion recognition abilities, the research data is considered scarce and contradictory. What can generally be argued is that social cognition disturbances have been associated with cognitive changes and reduced quality of life [53]. There are studies that clearly support deficits at least in some aspects of the ToM in MCI patients [16,53,54] while others challenge this view [55]. Regarding emotion recognition ability, the data tends to support deficient performance in recognizing at least certain emotions—mostly negative ones—[56,57] while in very recent studies, a disturbed ability to recognize positive emotions—happiness and surprise—was also shown [58,59]. For the Theory of Mind abilities of adults with vascular aging factors, the results are bidirectional; we encountered studies that succeed in showing deficits in ToM [60,61] and others that have disproved this hypothesis [62]. In a relevant cross-sectional study, it was shown that adults with vascular aging display significantly lower performance compared to the healthy population, at least in some aspects of the ToM that are complex enough, especially in the recognition of sarcasm [54].

In addition to the specific and distinct deficits in MCI, in the neuropsychological evaluation, a short test that assesses the global cognitive status of the patients is routinely used [63]. The main advantage of assessing global cognitive status over individual cognitive functions is that it is sufficient to establish a diagnosis while, in addition, it evaluates the progression of neurodegeneration and can provide an insight into the stage of the patient’s cognitive status [64]. For MCI especially, global cognitive decline is an important evaluation criterion, and, indeed, the speed of decline predicts the course of the disease and the progression to the next clinical stage [64]. The exploratory interest regarding the general cognitive assessment of adults with vascular risk factors is recent, and there are not many relevant studies in the literature. Two very recent studies support global cognitive deficits in adults with vascular aging; going deeper, a correlation between global cognitive status and vascular risk factors such as glucose levels and smoking has been suggested [65]. Moreover, global cognition has been correlated to carotid-femoral pulse wave velocity without further affecting the executive functions [66].

At this point, it is worth mentioning that Alzheimer’s disease progression also correlates with “Frailty” [67]; what connects the two pathological categories, beyond a common symptomatology—low physical activity, slow walking speed, self-reported exhaustion, and weight loss—are also some factors that affect the development of both of them, which has to do with vascular aging; for example, inflammatory status, co-morbidities like hypertension, diabetes or heart disease, and genetic factors can function as a substrate that can lead from vascular risk conditions to the development of Alzheimer’s disease [67]. Hence, an early detection of subclinical changes in molecular, cellular, and physiological level is proposed to delay “Frailty” and possibly Alzheimer’s pathology [68].

Taking into consideration the aforementioned theoretical background, the present study aimed to compare the cognitive and social cognition’s trajectories of two groups of people who may suffer from subtle or more severe cognitive impairment related to vascular pathology and neurodegeneration: (a) community dwelling people bearing vascular risk factors (VRF group) and (b) people bearing vascular risk factors and diagnosed with mild cognitive impairment (MCI), mostly with multi-domain amnestic MCI. As regards the comparison of the trajectories of the two groups, we hypothesized (a) that adults diagnosed with VRF + MCI will underperform in almost all assessments as compared to the group of adults with VRF only.

As regards cognitive and social cognition’s trajectories of VRF + MCI patients, based on the theory, there was the hypothesis (b) that the progression of MCI would be reflected in almost all the measurements of cognition and social cognition included in the extended neuropsychological battery of this study.

On the other hand, regarding the group of adults with VRF (without an MCI diagnosis), there was the hypothesis (c) that complex executive functions and working memory capacity would deteriorate. No specific hypothesis was formulated for social cognition due to the lack of clear previous findings.

## 2. Materials and Methods

### 2.1. Design

**Figure 1:**
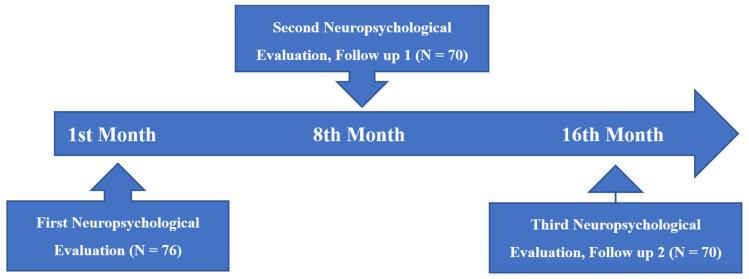
Timeline of the study assessment.

In this study, executive functions, Theory of Mind abilities, and memory capacity were assessed with extensive neuropsychological tests in two groups of participants: (a) people bearing vascular risk factors and (b) people with vascular risk factors plus a diagnosis of mild cognitive impairment. All participants were examined three times at the Health Center in Katerini, Greece, with a time gap of about 8 months (Figure 1). The first author completed the process of allocating participants in each group based on their medical examinations and diagnoses, medication, comprehensive medical history, and neuropsychological tests. The main neuropsychological assessment, which was administered three times, was divided into three sessions and lasted approximately two and a half hours.

### 2.2. Procedure

The evaluations were done in their entirety in the morning hours, in a quiet place without distracting stimuli. Prerequisites for the evaluation were an adequate night’s sleep; the participants were rested and had no physical discomfort (for example, fever, headache, or back pain) on the day of the evaluation. Examinees were given breaks when they felt they needed them. The neuropsychological testing was always done by the same examiner. As far as the order of administration is concerned, different versions of this battery were designed to avoid order effects.

### 2.3. Ethics

For the purposes of the study, the participants provided written informed consent at the time of their first visit, agreeing to their volunteer participation and their withdrawal at any time without providing any reason and without cost. The protocol of the study was approved by the Scientific and Bioethics Committee of the Greek Association of Alzheimer’s Disease and Related Disorders (Scientific Committee Approved Meeting Number: 25/21-06-2016) and followed the principles outlined in the Helsinki Declaration of 1975, as revised in 2008. Moreover, the study was approved by the Hellenic Data Protection Authority; having reviewed the research protocol, they confirmed that all ethical guidelines for research on human subjects were followed (License number: 1971). The names of the examinees were known only to the examiner and number codes were used for the further steps of the study; the researchers protect all the data in the Laboratory of Psychology, School of Psychology, AUTh, and maintain their complete anonymity.

### 2.4. Participants

The participants in the present study were a total of 70; 35 belonged to the group of VRF + MCI and 35 to VRF group. Six participants who did not complete all assessments were ultimately excluded from the study. The two groups did not differ significantly in age [t (70) = 0.853 *p* > 0.05], years of education [t (70) = −0.384, *p* > 0.05], and gender [χ2 (0.788) = 0.072, *p* > 0.05].

All participants were outpatients of the Health Center Clinics in Katerini. They were diagnosed in the last six months, and their health progress was regularly monitored by pathologists, general physicians, a diabetologist (in the case of VRF), and a neurologist (in the case of VRF + MCI). The sample did not comprise adults with mood and/or anxiety disorders, neurological disorders of any type, dementia of any type, patients diagnosed with cancer within the last five years, adults after stroke, myocardial infarction and cardiac instabilities, or patients having undergone any other surgery within the last five years. Adults with mental and/or psychiatric diseases and adults with alcoholism and/or drug use disorders were excluded from the study sample. All participants were screened for depressive symptomatology with the help of the Geriatric Depression Scale-15 [69,70], and persons with scores > 6 were excluded from the study. To assess the ability of simple and complex sentence comprehension, we used the subscale “auditory perception” from the Boston Diagnosing Aphasia Examination [71].

VRF + MCI group. The first group consisted of 35 adults (9 men and 26 women, Mean Age = 71.2, SD = 5.18) diagnosed with MCI during the last year. Their years of education ranged from 6 to 16 (Mean years of education = 10.4 S.D. 2.66). They all suffer from MCI of amnestic type with accompanying vascular risk factors for which they are taking the appropriate medication; therefore, it was a heterogeneous group with diverse etiologies for their cognitive decline, such as amyloid and vascular pathology. The inclusion criteria were based on the DSM-5 criteria for Mild Neurocognitive Disorders (American Psychiatric Association, 2013). Besides the neuropsychological assessment, their diagnosis was supported by neurological examination and blood tests. The inclusion criteria included (a) a diagnosis of Minor Neurocognitive Disorders according to the DSM-5 and (b) a 1.5 standard deviation (SD) below the normal mean in at least one cognitive domain, according to the neuropsychological tests. In addition, to support the diagnosis of MCI, a neuropsychological battery was administered which is presented in detail in Tsolaki et al. (2017) [72]

VRF group. The second group consisted of adults bearing Vascular Risk Factors with no diagnosis related to cognitive decline. They were under medical supervision and medication due to VRF (n = 10 men and 25 women, Mean Age = 70.1 SD = 5.46). Their blood test results from the previous six months showed at least one of three common VRF (hypertension, hyperlipidemia, diabetes mellitus). Exclusion criteria comprised diagnosis of MCI or dementia of any type, and all criteria described above. Their educational level ranged from 6 to 16 years (Mean years of education = 10.7 S.D. 2.93).

## 3. Measures

### 3.1. Main Neuropsychological Assessment

#### 3.1.1. Global Cognitive Status

The Montreal Cognitive Assessment was used to assess global cognitive status [73]. It was validated as a highly sensitive tool for early detection of MCI; MoCA accurately and quickly assesses short-term memory, visuospatial abilities, executive functions, attention, concentration and working memory, language, and orientation to time and place. For the purposes of the present study, its Greek version was used [74].

#### 3.1.2. Measures of Executive Functions

To assess initiation, inhibition, switching, and monitoring, the following tools were chosen: the Trail Making Test A & B by Reitan (1955), [75], Greek adaptation [76]; the Design Fluency Test from the Delis-Kaplan Executive Function System-D-KEFS [77], Greek adaptation [53].

Color-Word Interference Test: It consists of four conditions; the first is in essence color naming, while the second is reading ability, the third condition assesses inhibition ability while the last and more complex condition is evaluating inhibition/switching. Performance is measured by the completion time on each of the four trials; in addition, incorrect answers are counted and evaluated.

Trail Making Test A & B: Both parts of the Trail Making Test consist of 25 circles. In Part A, the circles are numbered 1–25, and the patient should draw lines to connect the numbers in ascending order. In Part B, the circles include both numbers (1–13) and letters (A–L); the patient draws lines to connect the circles in an ascending pattern, but with the added task of alternating between the numbers and letters. In the present study, the completion times of the two conditions were used as the variables of interest.

Design Fluency Test: It measures the examinee’s ability to draw as many different designs as possible in 60 s. The examinee is presented with rows of boxes, each containing an array of dots, and are asked to draw a different design in each box using only four lines to connect the dots. In condition 1, Filled Dots, the response boxes include only filled dots, and the examinee is asked to create the designs connecting those dots. In condition 2 (inhibition), Empty Dots Only, the response boxes contain both filled and unfilled (empty) dots. The examinee is asked to connect only the empty dots and to inhibit the previous response of connecting the filled dots. In condition 3 (switching), the boxes include both filled and unfilled dots. The examinee is asked to draw the designs by alternately connecting the filled and empty dots. The ranking scale is as follows: 1 point for each correct design and 0 points for designs that do not fit or are repeated. Incorrect designs are those for which the participant used more or less than 4 lines to draw, as those were the specific instructions for each condition that have not been followed. In the present study, the number of correct designs for each condition was used as the variable of interest.

#### 3.1.3. Measures of Memory

To assess short-term memory and working memory, we used the Digit Span from the WAIS III (Wechsler, 1995) [78]; translation and adaptation for the Greek population Stogiannidou 2014 [79].

Working Memory Index from the WAIS IV Gr: It measures short-term storage and processing (working memory capacity). It is comprised of the Digit Span Forward and Backward tasks. Condition 1 (forward) assesses the short-term retention, while Condition 2 (backward) attempts to assess the retention and processing of information (working memory capacity). In this task, the examinee is presented with the series of numbers in the order that they heard them in Condition 1 and is asked to repeat them in the reverse order (Condition 2). The length of each sequence of numbers increases as the examinee responds correctly. The number of correct recalls is calculated in the score, which corresponds to memory span. The set of the correct series of numbers that the participant recalls in each condition constitute the score.

Besides the short-term storage and working memory test mentioned above, a test measuring different aspects of retrospective long-term memory was also used. Specifically, the Doors and People test is designed to measure visual recognition, visual recall, verbal recognition, and verbal recall (Baddeley et al. 1994) [80], for Greek translation and adaptation Arampatzi & Masoura 2012 [81]. Four subtests (verbal/visual recall, verbal/visual recognition) were administered in the following order: immediate “verbal recall”, “visual recognition”, delayed “verbal recall”, immediate “visual recall”, “verbal recognition”, and delayed “visual recall”. In the first condition, the Verbal Recall—People subtest, the aim is for the examinee to memorize and remember the name and occupation of 4 people following the display of photographs. Both immediate recall and delayed recall are assessed. In the present study, only the score of the delayed assessment was used as variable of interest. The Visual recognition—Doors subtest includes 27 photos of colored doors (three of them are given as examples to practice; the rest are divided by 12 into two graded difficulty conditions). In addition to the 24 target doors, there are 81 distractor doors. The thing asked is to correctly recognize the target doors. In the next condition, Visual Recall—Shapes Subtest—the participants are shown 4 figures that directly draw them while in a second time are asked to recall them (delayed visual recall variable). In the last one, Verbal Recognition, participants are presented with 27 forenames and surnames (three of which are given as examples to practice, and the rest are divided by 12 into male and female names). The purpose of the trial is to recognize the correct surname as presented at the initial stage. The total correct responses for each condition were counted and presented here as scores and consist the variables of interest in present study. NOTE: for the recall conditions, only the scores for the delayed recall were used.

#### 3.1.4. Measures of Theory of Mind

The Awareness of Social Inference Test: The Simple Social Inference Test: The Social Inference—Minimal test (Mc Donald et al. 2003) [82] and for the Greek population Tsentidou et al. 2021 [55], includes two broad types of social exchanges; in the Sincere exchanges, the targeted speaker means what they are saying. In the Sarcastic exchanges, one of the speakers means the opposite of what they are saying. The aim is for the recipient to understand the real meaning of the words. The test presents two different subtypes of sarcastic exchanges. In the Simple Sarcasm exchanges, one of the participants is being sarcastic, but it is mainly by reading the paralinguistic cues that the viewer can discern the sarcasm. The dialogue in these scenes is identical to the dialogue in the Sincere Exchanges scenes. Therefore, if the viewer is unable to detect the sarcasm, they will read it as a sincere exchange and misinterpret both the intention of the speaker and the meaning. In Paradoxical Sarcasm exchanges, the dialogue between the two participants does not make sense, unless it is recognized that one of the participants is being sarcastic. In these scenes, if the viewer is unable to discern the sarcasm, it is difficult for them to make sense of the scene. Therefore, their decisions regarding speakers’ intentions, emotions, etc. are likely to be incorrect or bizarre. The number of correct responses for each category—Sincere exchange, Simple Sarcasm, Paradoxical Sarcasm—were the respective scores—variables used in the present study.

Emotion Evaluation Test: The EET (Mc Donald et al. 2006 [83]: In its Greek adaptation) is a visual tool designed for the clinical assessment of basic emotion recognition [55]. The test comprises a video of short scenes of actors interacting in everyday situations. The target actor in each scene exhibits one of the six basic emotions, or, in some scenes, the actor does not show any emotion (emotionally neutral condition). The participant watches each scene and is then asked to name the emotion they believe the actor was exhibiting from a multiple-choice array. The test consists of 28 scenes comprising four examples of each of the six emotional states and one non-emotional state [55]. In the present study, the variables as obtained from the SEM model from the adaptation of Moraitou et al. (2013) were used [84]; hence, the variables used were three: the total Emotion Recognition variable, the “Happiness” variable, and the “Neutral condition” variable.

## 4. Statistical Analysis

The data analysis was conducted in SPSS version 26 (IBM Corp., Armonk, NY, USA, 2016) [85]. The analyses carried out were (a) mixed-measures ANOVA, (b) repeated measures ANOVA, (c) ANOVA. The aim of the analyses was to compare the performance between the two groups and, at the same time, the performance of each group in the three different time measurements. Mauchly’s test of sphericity for the assessment of within-subject factor and Greenhouse-Geisser was used for the correction of sphericity violations. The Box’s Test was used for the assessment of the equivalence of covariance matrices, Levene’s test was used to assess the equality of variances, and Partial eta-squared (η2) was used for the estimation of the effect size. The Scheffe test was adopted for post hoc comparisons.

### 4.1. Results

Before the presentation of the results, it should be mentioned that the Trail Making Test A, Color—Word Interference Test, and Emotion Evaluation Test are not included in the statistical analysis presentation below since no significant findings were revealed from their administration. The Table 1 that follows shows M and S.D. for each one of the other tests in each time condition.

#### 4.1.1. Global Cognitive Status

**Figure 2:**
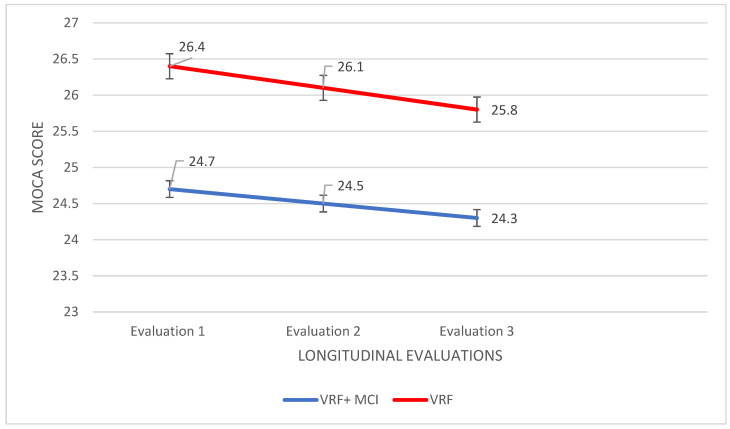
Performance of people with VRF and people with VRF + MCI in MoCA in the three times of assessment.

Montreal Cognitive Assessment: initially, a 2Χ3 mixed design ANOVA was performed with group (VRF + MCI & VRF) as the between-subjects factor and the time of assessment (three times) as the within-subjects factor. A significant main effect of the group was found, F (112,9,1) = 40.741, *p* < 0.0001, η2 = 0.375 as well as a significant main effect of the time of assessment, F (90487,2) = 7714, *p* < 0.0001, η2 = 0.102. Moreover, a significant group × time of assessment interaction was found F (67.000,2) = 8.812 *p* < 0.0001, η2 = 0.208. Subsequently, an analysis of variance was applied with the participant group as the independent variable and performance in each of the three times of assessment as the dependent variables. According to Pillai’s trace, the group effect was found to be significant, V = 0.999, F (66.000,3) = 15,665, *p* < 0.0001, η2 = 0.999. For the first time of measurement F (51429,1) = 36,537, *p* < 0.0001, η2 = 0.350; in the second time F (48057,1) = 31,353, *p* < 0.0001, η2 = 0.316; and in the third time F (18514,1) = 8503, *p* = 0.005, η2 = 0.111. VRF + MCI patients had worse performance in MoCA compared to VRF group in all evaluations, I-J = −1714, I-J = −1657, I-J = −1209 (Figure 2).

For a further analysis of the time effect, a repeated measures ANOVA was used for each group separately. For VRF + MCI patients, no significant results emerged. However, for the VRF group, performance gradually worsened: F (10314,2) = 14.600, *p* < 0.0001, η2 = 0.300. The Scheffe post hoc comparisons showed that in the third assessment, their global cognitive status was found to be significantly worse, I-J = −1057, *p* < 0.0001, as compared to the first time of assessment (Figure 2).

#### 4.1.2. Executive Functions

Trail Making Test B: Significant differences emerged from the B condition-completion time. A 2Χ3 mixed design ANOVA was performed with the group as the between-subjects factor and the time of assessment as the within-subjects factor. A significant main effect of the group was found, F (1937472,1) = 352.1, *p* < 0.0001, η2 = 0.838, and a significant group × time of assessment interaction, F (67.000,2) = 10,624, *p* < 0.0001, η2 = 0.241. Subsequently, analysis of variance was applied with the group as the independent variable and performance in each of the three times of assessment as the dependent variables. According to Pillai’s trace, the group effect was found to be significant, V = 0.980, F (66.000,3) = 1098, *p* < 0.0001, η2 = 0.980. The third measurement resulted in a significant difference between the two groups, F (2.616,1) = 9.414, *p* = 0.003, η2 = 0.122. The VRF + MCI patients had worse performance in time completion compared to the VRF group, I-J = −12.229.

Design Fluency Test: A 2 (VRF + MCI, VRF) Χ3 (three times of assessment) mixed design ANOVA was performed as concerns the data of the condition A (filled dots) of the Design Fluency Test. A significant main effect of the group was found, F (11293,1) = 29211, *p* < 0.0001, η2 = 0.300, as well as a significant main effect of the time of assessment, F (28.319,2) = 9.455, *p* < 0.0001, η2 = 0.122. Moreover, there was a significant group × time of assessment interaction, F (67.000,2) = 8.032, *p* = 0.0001, η2 = 0.193. Subsequently, the analysis of variance was applied with the group as the independent variable and performance in each of the three times of assessment as the dependent variables. According to Pillai’s trace, the group effect was found to be significant, V = 0.358, F (66.000,3) = 12.271, *p* < 0.0001, η2 = 0.358. In particular, the second and the third measurement resulted in significant differences between the two groups, F (46.414,1) = 16.441, *p* < 0.0001, η2 = 0.197, and F (68.014,1) = 18.814, *p* = 0.001, respectively. The VRF + MCI patients had worse performance in total correct designs compared to the VRF group, I-J = −1.629 for the second measurement and I–J = −1.971 for the third measurement.

As regards time effects, a repeated measures ANOVA was applied to the data of each group. The VRF + MCI patients showed statistically significant differences in the responses produced in different times of assessment, F (24.352,2) = 6453, *p* = 0.003, η2 = 0.160. Specifically, they produced a smaller number of correct designs in the third time, as compared to the first, I-J = −1.486 *p* = 0.010, and to the second time, I-J = −1.400, *p* = 0.030. With regards to the VRF group, F (10.314,2) = 4.654, *p* = 0.010, η2 = 0.016, this group produced smaller number of correct designs the third time as compared to the second one, I-J = −1.057, *p* = 0.016.

The same procedure was followed for the condition B of the same test (empty dots). A significant main effect of the group was found, F (107,1) = 36.133, *p* < 0.0001, η2 = 0.347, as well as a significant main effect of the time of assessment, F (25.062,2) = 10.097, *p* < 0.0001, η2 = 0.129. Moreover, there was a significant group × time of assessment interaction, F (67.000,2) = 13.644, *p* < 0.0001, η2 = 0.289. Subsequently, the analysis of variance was applied with the group as the independent variable and performance in each of the three times of assessment as the dependent variables. According to Pillai’s trace, the group effect was found to be significant, V = 0.462, F (66.000,3) = 18.857, *p* < 0.0001, η2 = 0.462. The second and the third measurement resulted in significant differences between the two groups, F (120,9,1) = 41.767, η2 = 0.381 and F (91.429,1) = 30.349, η2 = 0.309, respectively, *p* < 0.0001. The VRF + MCI patients had worse performance in total designs compared to the VRF group: I-J = −2.629 for the second measurement and I-J = −2.286 for the last one.

For the further analysis of time effects, a repeated measures ANOVA was applied to the data of each group. For the VRF + MCI patients, statistically significant differences in the responses were produced in different times of assessment, F (32.142,2) = 9.508, *p* < 0.0001, η2 = 0.219. They produced a smaller number of correct designs in the last assessment compared to the first one I-J = −1.914, *p* < 0.0001. In regards to the VRF group, F (16.124,2) = 10.113, *p* < 0.0001, η2 = 0.229, the second time they produced more correct designs than the first, I–J = 1.114, *p* = 0.008, while the third time they achieved less correct designs than the second one, I-J = −1.229, *p* < 0.0001.

**Figure 3:**
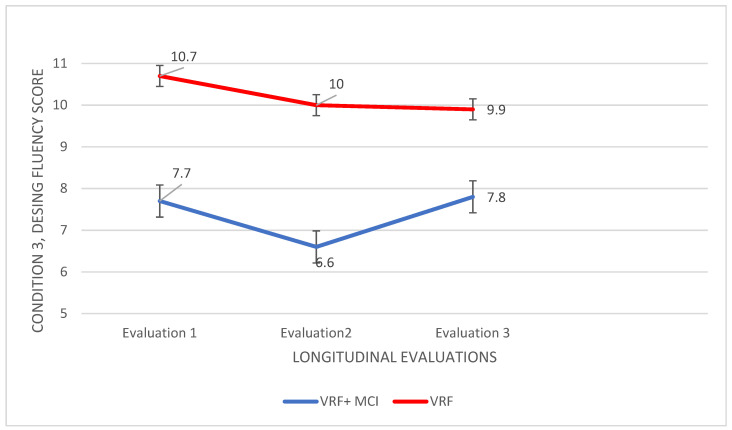
Performance of people with VRF and people with VRF + MCI in task/rule switching (Design Fluency: Condition C).

Regarding the condition C (filled—empty dots), the same statistical analysis was followed, and the results obtained are described in detail. A significant main effect of group was found, F (420,1) = 4.659, *p* < 0.0001, η2 = 0.637, as well as a significant main effect of the time of assessment, F (14.319,2) = 5.164, *p* = 0.007, η2 = 0.071. In addition, there was a significant group × time of assessment interaction, F (67.000,2) = 6.108, *p* < 0.0001, η2 = 0.154. An analysis of variance was applied to the data with the group as the independent variable and performance in each of the three times of assessment as the dependent variables. According to Pillai’s trace, the group effect was found to be significant, V = 0.657, F (66.000,3) = 42.153, *p* < 0.0001, η2 = 0.657. In all three assessments, the worst performance resulted for the group with the VRF + MCI diagnosis: I-J = −3.029, I-J = −3.343, I-J = −2.114 (Figure 3). 

For the further analysis of time effects, a repeated measures ANOVA was applied to the data of each group. For the VRF + MCI patients, a statistically significant difference in the responses produced in different times of assessment, F (14.181,2) = 4.199, *p* = 0.019 η2 = 0.110, was found. They produced a smaller number of correct designs in the last assessment compared to the second one, I-J = −1.143, *p* = 0.010. Regarding the VRF group, no significant differences in performance were noted.

#### 4.1.3. Memory

Forward and Backward Digit Span: For the first condition—Forward (short-term memory), a 2Χ3 mixed design ANOVA was performed. A marginally significant main effect of group was found, F (5.186,1) = 4.114, *p* = 0.046 η2 = 0.057, while a significant main effect of the time of assessment was also shown, F (7.390,2) = 10.109, *p* < 0.0001, η2 = 0.129. Moreover, a significant group × time of assessment interaction came up, F (67.000,2) = 11.172, *p* < 0.0001, η2 = 0.250. An analysis of variance showed no significant findings.

**Figure 4:**
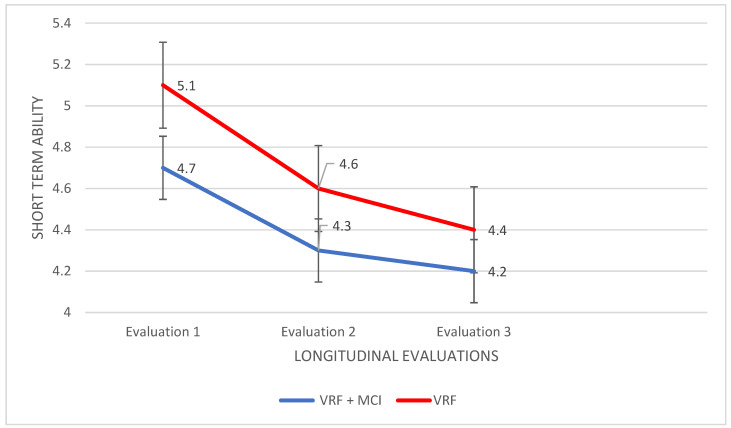
Performance of adults with VRF and adults with VRF + MCI in the Forward condition (short-term memory) of Digit Span Test.

In the next step, repeated measures ANOVA was applied to the data of each group. For the VRF + MCI patients, no significant results were found. However, in the VRF group, performance differed in the measurement times, F (5.067,2) = 7.198, *p* < 0.0001, η2 = 0.175, with VRF to retain and repeat significantly fewer numbers in the third time of assessment compared to the first, I-J = −0.743, *p* = 0.004 (Figure 4).

**Figure 5:**
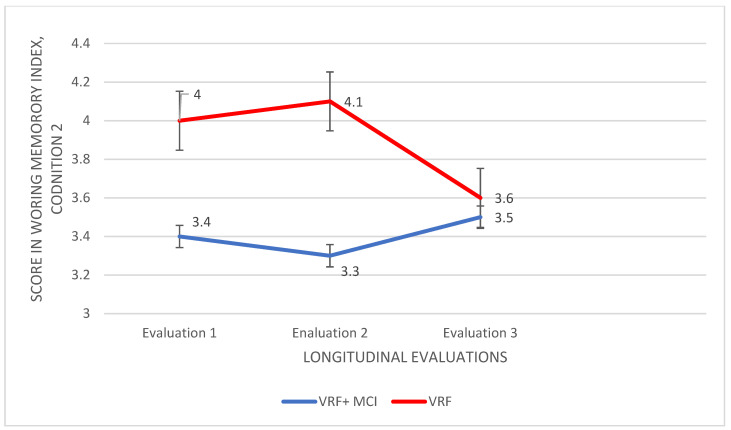
Performance of adults with VRF and adults with VRF + MCI in the Backward condition (working memory) of Digit Span Test.

For the second condition—Βackward (working memory), the same statistical procedure was followed. Therefore, a significant main effect of the group was found, F (2.860,1) = 3.851, *p* < 0.0001, η2 = 0.983, as well as a main effect of the time of assessment, F (1.671,2) = 7.704, *p* = 0.001, η2 = 0.102; no significant group × time of assessment interaction emerged. An analysis of variance showed significant results, V = 0.275, F (66.000,3) = 8.338, *p* < 0.0001, η2 = 0.275, at the first measurement, F (5.714,1) = 13.102, *p* = 0.001, η2 = 0.162, and at the second one, F (9.657, 1) = 25.538, *p* < 0.0001, η2 = 0.273. Adults diagnosed with VRF + MCI performed worse compared to the VRF group at both time points, I-J = −0.571 and I-J = −0.743 respectively. However, at the third assessment, the performance of the two groups was found almost the same (see Figure 5).

Subsequently, a repeated measures ANOVA was applied to the data of each group. The VRF + MCI patients showed no significant results. Nevertheless, for the VRF group, the performance differed in the time measurements, F (2.067,2) = 9.246, *p* < 0.0001, η2 = 0.214. The VRF adults could retain and repeat less numbers in the third time of assessment compared to the second one, I-J = 0.457, *p* = 0.004 (Figure 5).

**Figure 6:**
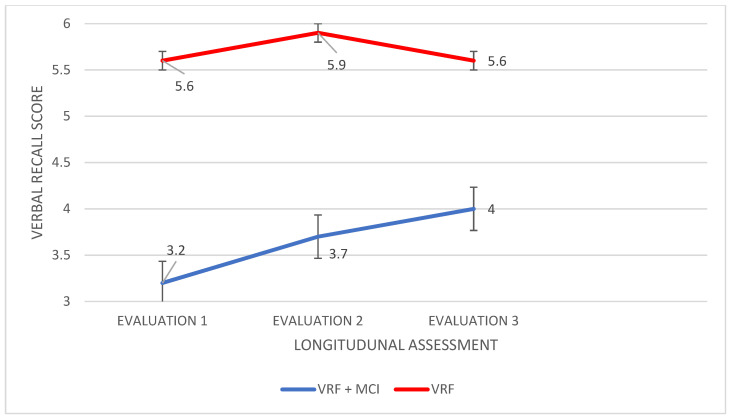
Performance of people with VRF and people with VRF + MCI in Delayed Verbal Recall (long-term memory).

Retrospective long-term memory (Doors and People test): Significant results emerged only for the Verbal Recall subtest and only in terms of the difference between groups. According to Pillai’s trace, the group effect was found to be significant, V = 0.495, F (66.000,3) = 21.585, *p* < 0.0001; the performances differed in all three measurements: F (103,2, 1) = 40.390, *p* < 0.0001, η2 = 0.373 for the first assessment; F (82.514,1) = 45.970, *p* < 0.0001, η2 = 0.403 for the second one; and F (44.800,1) = 26.458, *p* < 0.0001, η2 = 0.280 for the last measurement. The VRF + MCI patients had worse performance in Verbal Recall compared to the VRF group in all assessment times, I-J = −2.429, I–J = −2.171, and I-J = −1.600 (Figure 6).

#### 4.1.4. Theory of Mind

**Figure 7:**
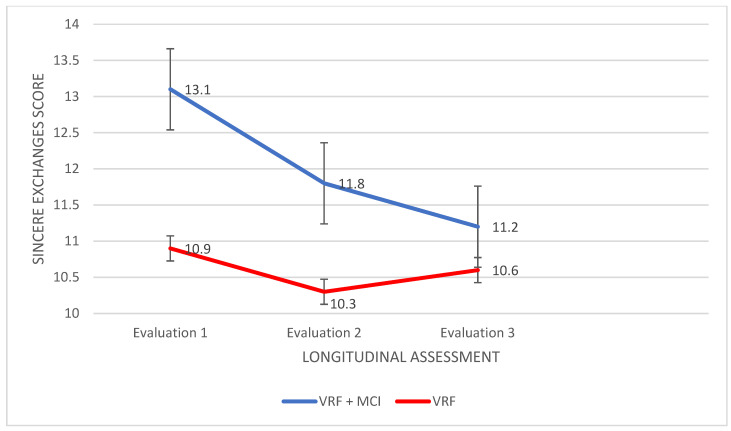
Performance of adults with VRF and adults with VRF + MCI in Sincere Exchange condition of the TASIT (ToM abilities).

The Awareness of Social Inference Test: The Simple Social Inference Test: A 2 (VRF + MCI, VRF) Χ3 (three times of assessment) mixed design ANOVA was performed as regards the sincere exchanges (control condition). A significant main effect of group was found, F (27.109,1) = 65.888, *p* < 0.0001, η2 = 0.990, as well as a significant main effect of the time of assessment, F (24.776,2) = 5412, *p* = 0.005, η2 = 0.074. In addition, there was a significant group × time of assessment interaction, F (67.000,2) = 4.691, *p* = 0.012, η2 = 0.123. Subsequently, an analysis of variance was applied with the participant group as the independent variable and performance in each of the three times of assessment as the dependent variables. According to Pillai’s trace, the group effect was found to be significant, V = 0.175, F (66.000,3) = 9.678, *p* < 0.0001, η2 = 0.175. Significant differences between the two groups emerged in the first and the second assessment: F (80.357,1) = 14.436, *p* < 0.0001, η2 = 0.175, and F (43.214,1) = 13.172, *p* = 0.001, η2 = 0.162, respectively. The VRF + MCI patients had higher performance in Sincere scenes as compared to the VRF group in both evaluations, I-J = 2.143, I-J = 1.571 (Figure 7).

In the next step, repeated measures ANOVA was applied to the data of each group. For VRF + MCI patients, significant results emerged: F (31.229,2) = 6.157, *p* = 0.003, η2 = 0. 153. In particular, in the second assessment VRF + MCI had lower performance compared to the first assessment, I-J = −1.229, *p* = 0.009. The same was found as regards the third compared to the first assessment, I-J = −0.857, *p* = 0.003. No significant results emerged for the VRF group.

As regards Simple Sarcasm understanding, a significant main effect of the group was found, F (114.4,1) = 30.100 *p* < 0.0001, η2 = 0.307, as well as a significant main effect of the time of assessment, F (30.406,2) = 10.036, *p* < 0.0001, η2 = 0.129. Moreover, there was a significant group × time of assessment interaction, F (67.000,2) = 8.912, *p* < 0.0001, η2 = 0.210. Subsequently, an analysis of variance was applied with the participant group as the independent variable and performance in each of the three times of assessment as the dependent variables. According to Pillai’s trace, the group effect was found to be significant, V = 0.389, F (66.000,3) = 14.025, *p* < 0.0001, η2 = 0.389; differences between the two groups emerged in the third time of assessment, F (151,1) = 40.725, *p* < 0.0001, η2 = 0.372. The VRF + MCI patients had worse performance in Simple Sarcasm scenes compared to the VRF group, I-J = −2.943.

**Figure 8:**
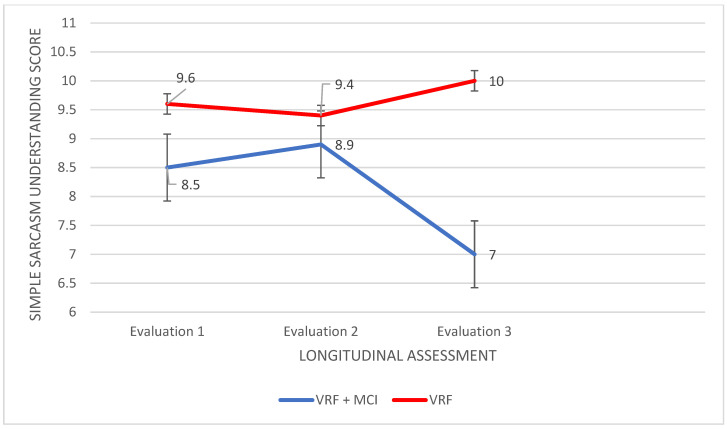
Performance of people with VRF and people with VRF + MCI in Simple Sarcasm understanding (ToM abilities).

In the next step, a repeated measures ANOVA was applied to the data of each group. For the VRF + MCI patients, significant results emerged: F (35686,2) = 11.202, *p* < 0.0001 η2 = 0.048. In the third assessment, the VRF + MCI patients had lower performance compared to the first assessment, I-J = −1.514, *p* = 0.002. The same was found regarding the third compared to the second assessment, I-J = −1.914, *p* < 0.0001 (Figure 8). On the other hand, for adults bearing VRF, no significant results were found.

As concerns Paradoxical Sarcasm, significant results were found only in terms of the difference between groups. According to Pillai’s trace, the group effect was found to be significant, V = 0.222, F (66.000,3) = 6.287, *p* = 0.001. The performances differed in the second, F (19.557, 1) = 6.835, *p* = 0.014, η2 = 0.086 and in the third assessment, F (38.629,1) = 12.209, *p* = 0.001, η2 = 0.152. The VRF + MCI patients had worse performance in Paradoxical Sarcasm as compared to the VRF group in both assessments, I-J = −1.057 and I–J = −1.486, respectively.

## 5. Discussion

The aim of the present study was to compare cognitive and social cognition’s trajectories of two groups of people who may suffer from subtle or more severe cognitive impairment related to vascular pathology and neurodegeneration: (a) community dwelling people bearing vascular risk factors (VRF group) and (b) people bearing vascular risk factors and diagnosed with mild cognitive impairment (MCI), mostly with multi-domain amnestic MCI.

As observed from the detailed presentation of the statistical analysis, clear significant results regarding the differential diagnosis of the two groups and specific significant findings regarding the development of pathology in each group have arisen.

### 5.1. Cognitive Abilities’ Decline in People with VRF

From the variety of selected tools and the assessment of the VRF and VRF + MCI groups in three times interval, it became apparent that different tests succeed in ‘diagnosing’ cognitive deterioration in each group. In detail, the adult group with VRF displays a downward trajectory on the performance in MoCA and in short-term retention, with the third time assessment to be significantly lower compared to the first. Hence, the two cognitive abilities that seem to deteriorate in this group are global cognition and short-term memory storage. Recently, Yuxing Kuang at al. 2022 [86] confirmed with imaging methods the correlation of glucose metabolism and insulin resistance with impaired functional connectivity, which is associated with MoCA performance. Moreover, the same findings have been supported by Michailidis et al. 2022 [87], who reviewed and discussed common pathophysiological mechanisms between Alzheimer Disease and type 2 diabetes mellitus; this review study suggests the frequent assessment of the cognitive status of patients with type 2 diabetes mellitus as it is an important risk factor for the onset of AD dementia.

Moreover, it has already been shown that vascular aging causes vascular gray matter injuries [88]; structural damage is observed in regions like hippocampal CA1 and layer 3 and 5 of the cerebral cortex [89], areas directly linked to memory function.

Thus, the present study shows that community dwelling adults of advancing age with vascular risk factors (at least one of them) could suffer from incipient cognitive decline due to vascular pathologies, which can be “captured” in a developmental trajectory of about 1.5 year. This means that they should be assessed at least every 2 years to detect potential cognitive impairment as soon as possible. At this stage of impairment, it is possible to be able to reverse cognitive decrements via simple changes in lifestyle.

Another interesting finding for this group is that the performance in the Backward condition from the Digit Span Test is almost the same with that of people with the VRF + MCI in the third time assessment. In other words, besides global cognition and short-term memory, working memory seems to suffer too. However, the trajectory of working memory decline is somehow different -delayed and sharp- as compared to the trajectories of the other two cognitive abilities. In a recent review [90] it has been extensively discussed the association between cardiovascular health and working memory capacities. Two specific vascular factors have already been noted: arterial elasticity and fitness [91]. The relationship between fitness and working memory was found to be mediated by the concentration of a neuronal metabolite; that is deemed important for the viability of neurons in the brain. Several other ‘reasons’ have been elucidated that attempt to explain the association between working memory capacity and vascular aging: atherosclerosis, cerebral oxygenation-perfusion, small subcortical infarcts and lacunes, and cortical superficial siderosis [92,93]. Besides the underlying brain substrate, a specific low level of short-term storage might function as a prerequisite for the relatively sharp decline of working memory in the VRF group compared to short-term storage decline.

In sum, at the clinical level, it would be useful to assess at least every 2 years community dwelling people bearing vascular risk factors, with short measures of (a) global cognition, (b) short-term retention, and (c) working memory capacity.

### 5.2. Social Cognition Abilities’ Decline in People with VRF + MCI

Regarding the adults with VRF + MCI, it seems they show a more general decline in cognition that lies in more functions, which is mainly and more clearly reflected in their performance on more complex tasks such as the ToM tests. In the Sincere exchanges condition as well as in the Simple Sarcasm condition (ToM), their performance was significantly poorer in the third measurement compared to previous time measurements. In the ToM deficits disruptions to broader neural networks are likely to be implicated [94] prominent atrophy and tau deposition has been observed in the hippocampus and amygdala, and this may contribute to deficits in social cognition [95,96]. Volumetric changes in limbic structures—the amygdala and hippocampus, lesions evident in MCI—have been associated with impaired performance in the ToM tasks [94]. Executive functioning impairments such as those found for initiation, inhibition, and switching (Design Fluency A, B, C) abilities might also contribute to ToM decline in the VRF + MCI group, since frontal lesions are well-established in MCI patients.

Therefore, it seems important for the evaluation of the trajectory of MCI pathology in patients with a vascular risk substrate to assess some relatively complex aspects of the Theory of Mind such as indirect speech understanding (e.g., sarcasm, humor, and irony comprehension). On the other hand, simple socio-cognitive abilities such as emotion recognition cannot seem to help to “capture” the trajectory of cognitive decline in the VRF + MCI pathology [97].

### 5.3. Cognitive Abilities Which Differentiate People with VRF from People with VRF + MCI 

Regarding group differentiation, based on the findings, there are three tests that can clearly and steadily “capture” the differences in the prototypes of cognitive impairment in the two pathologies. The MoCA differentiates the two groups in all three times of their assessment with the lowest score consistently recorded for the VRF + MCI adults. The same is true for the last and most demanding condition of the Design Fluency test as well as for delayed verbal recall. Hence, global cognitive status, complex executive functions—cognitive flexibility such as visuospatial task/rule switching, and retrospective long-term memory (episodic) seem to be the three cognitive “areas” that could more clearly than others indicate that brain pathology related to neurodegeneration (most often, AD pathology) has been developed in a substrate of vascular pathologies.

Describing Alzheimer’s from a more biological perspective, it is characterized as a disease that accumulates abnormal entities: β-amyloid deposition, pathological tau, and neurodegeneration [98]. It has been shown that cortical tau tangles appear much earlier and have a progressive course; first, they are observed in medial temporal lobe -including the entorhinal cortex and hippocampus, then they advance toward limbic structures, and they finally spread to frontal, parietal, and temporal cortical regions, as well as to subcortical structures [99], differentiating in this way their effect on cognitive skills at the time of establishment and during the course of the disease [100]. It has also been demonstrated that metabolic syndrome-effect of cardiovascular risk factors brings about onset and progression of cerebral small vessel dysfunctions by altering the structure and function of the blood vessels, which can lead to mild bleeding, white matter damage, and brain atrophy, secondarily causing decline in cognitive functions [101,102,103,104,105,106].

There is a large body of literature discussing the impairment of adults diagnosed with MCI in both executive and memory functioning. Reviewing very recent literature, a 2021 study [107] states that the core deficits in MCI patients, such as cognitive flexibility decrements, are significantly correlated with functional mobility hinting at a correlation between cognitive and motor flexibility that affects the everyday life of these adults. It is already known that executive functions are mediated from frontal lobes [108]. In this context, the association of the superior frontal, rostral middle frontal, lateral/medial orbitofrontal, and retrosplenial cortices with switching abilities has already been shown. While in the same areas, changes of the white matter tract radial and mean diffusivity have been recorded as characteristic of adults with MCI as well [109]. Hence, task/rule switching as a complex executive function might decline due to its specific brain substrate that is affected by mixed pathologies. However, at this point it should be mentioned that it is visuospatial switching the function that suffers and not other types of switching, since the respective condition of the Color-Word Interference test did not result in significant findings in the present study.

It has been argued that an important landmark for MCI diagnosis is a deficit in episodic memory [110]. Indeed, in terms of episodic memory in MCI, the research data is considerable and tends to agree with each other. Episodic memory engages several brain regions: hippocampus, parahippocampal gyrus, entorhinal cortex, posterior cingulate gyrus, precuneus, cuneus, middle frontal gyrus, middle temporal gyrus, and lingual gyrus [111]. There is degeneration in all these regions and this results to a deterioration of episodic memory itself, which has been related with the progression of Alzheimer’s disease [112]. In a 2022 meta-analysis [113] that reviewed 21 studies with imaging techniques, it is supported that the degeneration of specific areas that support the function of episodic memory, such as the parahippocampal gyrus, precuneus, posterior cingulate gyrus, cuneus, middle temporal gyrus, middle frontal gyrus, lingual gyrus, and thalamus, is so directly related to the deterioration of patients that it could itself be a way of assessing the stages of MCI and by extension the transition to Alzheimer’s disease dementia [114]. Moreover, from another study using “Doors and People” test, has became clear that episodic memory tests are a valid and sensitive tool for distinguishing Alzheimer’s disease dementia from an aMCI, and also early aMCI from late aMCI [34].

Moreover, as regards chiefly global cognitive status, the reduction that occurs in processing speed in MCI [ (Neuropsychological Impairments and Their Cognitive Architecture in Mild Cognitive Impairment (MCI) with Lewy Bodies an MCI-Alzheimer’s Disease) can explain some part of it. The executive deficits and long-term memory deficits could also function in the same direction since tests such as MoCA include relevant subtests.

## 6. Conclusions

In conclusion, our findings indicate that specific neuropsychological tests that reflect specific cognitive and socio-cognitive abilities can differentiate between people bearing vascular risk factors and people with both VRF and mild cognitive impairment and, at the same time, are able to assess the cognitive and socio-cognitive trajectories of the two groups. Global cognitive status and short-term memory are the main cognitive abilities that decline in community dwelling people bearing VRF while in people with both VRF and MCI, it seems that the assessment of Theory of Mind abilities can better capture their further impairment. Global Cognitive Status, task/rule switching function, and long-term memory (delayed verbal recall) are revealed as the abilities that clearly and steadily differentiate VRF people with and without MCI.

Regarding the hypotheses of the study, in terms of the differentiation between the two groups and the expected poorer performance of the VRF + MCI patients compared to the VRF adults (hypothesis a), the hypothesis was confirmed for a major part of the selected neuropsychological tests. As has already been mentioned, the VRF + MCI adults achieved poorest performance in almost all tests in different time measurements. However, only three tests resulted in clear findings for all time assessments: MoCA (global cognition), condition C (visuospatial switching) of the Design Fluency Test, and delayed verbal recall (long-term, episodic memory) of the Doors and People test.

In terms of the cognitive and social cognition’s trajectories of the VRF + MCI patients, there was the hypothesis (b) that the progression of MCI would be reflected in almost all the measurements of cognition and social cognition included in the extended neuropsychological battery of this study. This hypothesis has been confirmed since; as discussed at length, participants in this group showed a generalized impairment across most cognitive skills: Design Fluency, Working Memory and ToM abilities.

On the other hand, regarding the group of adults with VRF (without MCI diagnosis), there was the hypothesis (c) that complex executive functions and working memory capacity would deteriorate. No specific hypothesis was formulated for social cognition due to the lack of clear previous findings. The results for this group indicated progressively deficient performance in general cognitive status—MoCa—and Working Memory.

### Limitations and Future Research

The study has some limitations. The first author, who was the only examiner, was not blinded to the clinical diagnosis of the three groups. In a future study, it would be useful to add more assessments so that the critical time interval for transition to more severe pathology can be supported clearly. Moreover, specific vascular risk factors or vascular pathologies should be examined separately for their relations to cognitive impairment longitudinally. Specific types of MCI should also be examined in the same vein. A larger number of participants could ensure a more reliable picture of the cognitive skills affected in the progression of vascular aging to neurodegeneration. It is additionally noted that the participants of this study who comprised the VRF + MCI group have not been investigated for biomarkers of Alzheimer’s Disease, a fact that could affect the results. The hypothesis of coexistence of “Frailty” in the participants was also not assessed. In the studies already carried out by our research group, the use of imaging methods and Cerebrospinal Fluid biomarkers has been added.

## Figures and Tables

**Table 1:** Mean Score and Standard Deviation of the neuropsychological tests in the three times of assessment.

		EVALUATION 1	EVALUATION 2	EVALUATION 3
**MOCA**				
	MCI	24.7 (SD 1.3)	24.5 (SD 1.4)	24.3 (SD 1.7)
	VRF	26.4 (SD 1.0)	26.1 (SD 1.0)	25.8 (SD 1.1)
**TRAIL TEST B**				
	MCI	88.9 (SD 16.8)	116.3 (SD 137.9)	102.9 (SD 22.3)
	VRF	87.2 (SD 11.8)	90.2 (SD 11.8)	90.7 (SD 7.6)
**DESIGN FLUENCY 1**				
	MCI	9.6 (SD 1.8)	9.5 (SD 1.8)	8.1 (SD 2.1)
	VRF	10.4 (SD 1.8)	11.1 (SD 1.4)	10.1 (SD 1.6)
**DESIGN FLUENCY 2**				
	MCI	10.6 (SD 1.8)	9.0 (SD 1.9)	8.1 (SD 1.9)
	VRF	10.5 (SD 2.0)	11.6 (SD 1.4)	10.4 (SD 1.4)
**DESIGN FLUENCY 3**				
	MCI	7.7 (SD 1.7)	6.6 (SD 1.8)	7.8 (SD 1.9)
	VRF	10.7 (SD 1.6)	10.0 (1.4)	9.9 (SD 1.7)
**SHORT-TERM MEMORY**				
	MCI	4.7 (SD 0.89)	4.3 (SD 0.86)	4.2 (SD 0.83)
	VRF	5.1 (SD 1.1)	4.6 (1.8)	4.4 (SD 0.81)
**WORKING MEMORY**				
	MCI	3.4 (SD.0.50)	3.3 (SD 0.49)	3.5 (SD 0.50)
	VRF	4.0 (SD 0.78)	4.1 (SD 0.71)	3.6 (SD 0.68)
**LONG-TERM MEMORY**				
	MCI	3.2 (SD 0.71)	3.7 (SD 0.91)	4.0 (SD 0.70)
	VRF	5.6 (SD 2.1)	5.9 (SD 1.6)	5.6 (SD 1.6)
**SINCERE EXCHANGE**				
	MCI	8.5 (SD 1.8)	8.9 (SD 1.4)	7.0 (SD 2.1)
	VRF	9.6 (SD 1.7)	9.4 (SD 1.7)	10 (SD 1.7)
**SIMPLE SARCASM**				
	MCI	13.1(SD 2.0)	11.8 (SD 1.6)	11.2 (SD 2.4)
	VRF	10.9 (SD 2.6)	10.3 (SD 1.9)	10.6 (SD 1.6)
**PARADOXICAL SARCASM**				
	MCI	10.0 (SD 1.9)	9.2 (SD 1.7)	8.9 (SD1.9)
	VFE	10.6 (SD 2.2)	10.2 (SD 1.7)	10.4 (SD 1.5)

## Data Availability

Not Applicable.
